# Earable ZEN: Development of an Earphone-Type Zazen Support Wearable System

**DOI:** 10.1155/2018/1838563

**Published:** 2018-12-11

**Authors:** Kazuhiro Taniguchi, Atsushi Nishikawa

**Affiliations:** ^1^Graduate School of Information Sciences, Hiroshima City University, 3-4-1 Ozukahigashi, Asaminami-ku, Hiroshima 731-3194, Japan; ^2^Faculty of Textile Science and Technology, Shinshu University, 3-15-1, Tokida, Ueda, Nagano 386-8567, Japan; ^3^Division of Biological and Medical Fibers, Institute for Fiber Engineering (IFES), Interdisciplinary Cluster for Cutting Edge Research (ICCER), Shinshu University, 3-15-1 Tokida, Ueda, Nagano 386-8567, Japan

## Abstract

Meditation has been included in mental health care and the treatment of hypertension and pain. Zazen is a method of entering the meditative state. We have carried out R&D on a wearable system (earable ZEN) that casually and simply allows the subject to perform zazen without going to a temple or even having a zazen monitor present. In zazen, it is important to prepare one's breathing and posture. The earable ZEN is comprised of an earphone-type sensor (ear sensor) for measuring the breathing and posture of the user, a miniature actuator (neck belt) for communicating disturbances in the breathing and posture of the user, and a microcomputer. In an evaluation experiment, disturbance in breathing was simulated as “deep mouth breathing” and disturbance in posture was simulated as “nodding”. The average *accuracy* value for seven healthy subjects wearing the earable ZEN was 99.9% for mouth breathing and 100% for nodding. In the same way, the average *precision* value was 98.7% for mouth breathing and 100% for nodding, with an average *recall* value of 97.1% for mouth breathing and 100% for nodding. None of the subjects considered the ear sensor and neck belt to be an obstacle to zazen.

## 1. Introduction

Meditation has been included in mental health care and the treatment of hypertension and pain [1–3]. Zazen is a method of entering the meditative state. Zazen involves concentrating the mind in a seated state [4–7]. In zazen, it is important to prepare your body (posture), your breathing, and your mind (spirit). In concrete terms, “preparing your body” refers to a posture in which you sit, by placing your right foot on your left thigh and your left foot on your right thigh, relaxing your shoulders and stretching your back. Additionally, tuck in your chin, so that your tongue touches the base of your front teeth, close your mouth gently, with no expression, your eyes half shut, and fix your gaze 1 m ahead of you. Place the back of one of your hands on the palm of the other hand, so that it covers the four fingers from your index finger to the little finger. The palms of both hands should be facing up. Place your left and right thumbs in a straight line so that their tips can touch. “Preparing your breathing” refers to performing abdominal breathing through your nose (do not breathe through your mouth) with your mouth closed. “Prepare your mind” refers to relaxing your mind. Here, repeatedly counting from 1 to 10 to yourself will help you to relax your mind.

In Japan, zazen is carried out in the “Zendo” meditation hall of a temple. In the Zendo, a supervisor, known as a “Jikijitsu” or “Jikido” moves around the Zendo during zazen and inspects those performing zazen. In case there are disturbances in the posture or breathing of those performing zazen, those performing the zazen are struck with a stick, known as a “Keikakusakurei” by the Jikijitsu or Jikido on either the right shoulder or the back. In zazen, the Keikakusakurei is considered to be a substitute for the bodhisattva, and as being struck with the Keikakusakurei has the meaning of “receiving the encouragement of the bodhisattva,” this is not considered to be corporal punishment. Sleepiness is one of the main causes of disturbances in breathing and posture. In zazen, to prevent sleepiness, it is determined that the eyes are kept half open. In the case of yawning (disturbances in breathing) seen when those performing zazen start to feel drowsy or movements such as shaking of the head up and down while seated (disturbances in posture) occur, they are struck by the Keikakusakurei of the Jikijitsu or Jikido.

We have carried out research and development on a wearable system (earable ZEN) that provides support to allow the practitioner to perform zazen casually and simply at home, without going to the temple, and on their own, without the presence of a Jikijitsu or Jikido. Until now, there have been many studies [8–12] investigating the heart rate and analysis of brain waves [13–24] during the meditative state of zazen or yoga. However, there has not been significant research and development into a wearable system for supporting meditation during zazen, as is provided by our study. In earable ZEN, an earphone-type sensor (ear sensor) monitors the breathing and posture of the person performing zazen in place of the Jikijitsu or Jikido. Additionally, if it is judged that there is a disturbance in the breathing or posture, a light vibration is applied to the neck of the user using an actuator, in place of the Keikakusakurei.

Earable ZEN does not evaluate the meditative state using brain waves (electroencephalogram: EEG) as seen in prior studies [13–24] instead monitors the breathing and posture of those performing zazen in lieu of a Jikijutsu or Jikido. In particular, earable ZEN detects incorrect breathing “performing deep mouth breaths (not breathing through the nose)” and incorrect posture “nodding (dozing)” when conducting zazen.

Until now, we have successfully carried out research and development on a hands-free interface that uses measurement results obtained from an ear sensor to measure meal times, number of chews, and tongue exercise [25–31]. The earable ZEN applies this R&D. A characteristic of the earable ZEN is that it can measure disturbances in breathing and posture with just one ear sensor using a simple mechanism. In this paper, we discuss the mechanism and evaluation results of the earable ZEN.

## 2. Materials and Methods

### 2.1. Hardware

**Figure 1:**
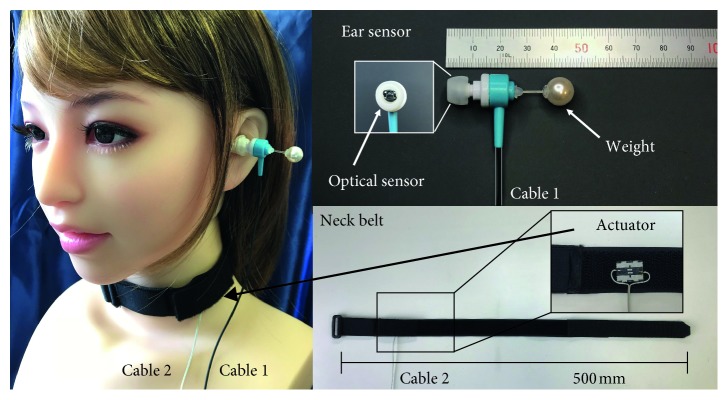
External diagram of the earable ZEN. The earable ZEN is used by attaching the ear sensor to the left ear and winding the neck belt around the neck. The ear sensor measures deep mouth breathing (movement simulating disturbance in breathing) and nods (movement simulating disturbance in posture). When mouth breathing or nods are detected, the actuator built into the neck belt is activated, and a light vibration is applied to the left side of the neck. This vibration communicates to the subject that “there was a disturbance in breathing or posture.” The neck belt has a two-layer structure, and the actuator is embedded within the first layer (surface layer) and the second layer. The photograph of the actuator in the figure has the first layer peeled away so the actuator can be seen. The ear sensor and neck belt can be attached while wearing a variety of clothing, such as formal zazen clothes (stole), a T-shirt, or a collared shirt.

**Figure 2:**
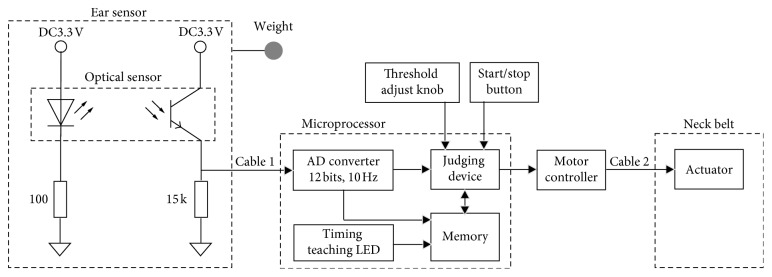
Block diagram of the earable ZEN. The earable ZEN is comprised of an earphone-type ear sensor, an actuator attached to a neck belt, an embedded microcomputer programmed in the C language, a start/stop button, and a threshold adjust knob. An optical sensor (LED and phototransistor) and weight are attached to the ear sensor. The microcomputer, based on the principles shown in Figure 3, where the variation in the measured values received from the ear sensor is greater than the *threshold* stated in Section 2.2, judges that “there was a disturbance in the breathing or posture” and activates the actuator attached to the neck belt. *Threshold* is obtained using the method described in Section 2.2, and this can be finely tuned using the threshold adjust knob. The earable ZEN can also start and stop operation using the start/stop button.

An external view of the earable ZEN is shown in Figure 1, and a block diagram is shown in Figure 2. As shown in Figure 1, with the earable ZEN, an ear sensor is attached to the left ear, and a neck belt is wound around the neck. The neck belt can be attached when wearing a variety of clothing, such as formal zazen clothes (stole), a T-shirt, or a collared shirt. The earable ZEN, by concentrating devices in the neck and left ear, enables the stole to be worn, and, as these do not get in the way when you are struck on the right shoulder with the Keikakusakurei, they can be used for actual zazen.

As shown in Figure 2, the earable ZEN consists of one ear sensor for measuring disturbances in the breathing or posture of the user, one miniature actuator for communicating the disturbance in breathing or posture to the user, and one microcomputer for judging that there has been a disturbance in the breathing or posture of the user based on the information from the ear sensor and controlling the actuator.

During zazen, you should breathe slowly through your nose and sit without moving your head or body. In this study, if the person performing the zazen (user) has a disturbance in breathing by accidentally performing mouth breathing or yawning, this disturbance in breathing is simulated as a “deep mouth breath”. Alternatively, if the posture is disturbed by nodding off or shaking of the head, this disturbance in posture is simulated as a “nod”. Hereafter, these yawns, nodding off, breathing, or posture disturbances are simulated as “mouth breathing or nod” disturbances in breathing or posture, and it is abbreviated to MN.

With the earable ZEN, changes in the shape of the ear canal due to the breathing of the subject, or shaking of the ear sensor itself due to nodding, are measured by the optical sensor built in to the ear sensor, and this measurement value is converted to a digital value by an analog-digital (AD) converter. When it is judged that “there is MN” by the judging device based on this AD converted value, the actuator attached to the neck belt is operated, and a slight 150 Hz vibration is applied to the left side of the subject's neck. The subject, based on this slight vibration, then becomes aware that there is a disturbance in their own breathing or posture.

**Figure 3:**
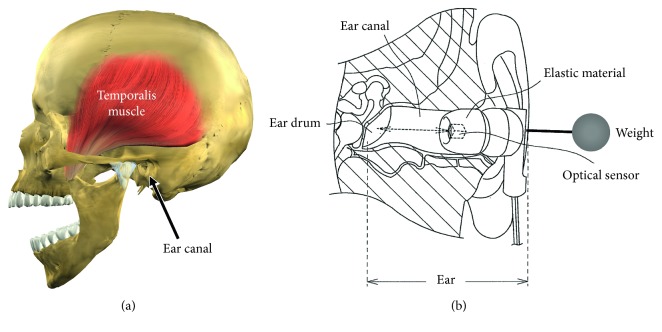
Measurement principles of the ear sensor. The ear sensor is used for the measurement of “deep mouth breathing” and “nods.” An optical sensor and weight are attached to the ear sensor. The optical sensor has one infrared LED and one phototransistor embedded, infrared light is irradiated on to the epidermis of the ear canal, and as the inverted light is received by the phototransistor, it is able to measure movements in the ear canal and shaking of the ear sensor. The opening and closing operation of the mouth when humans breathe through the mouth is achieved through the expansion and contraction of the temporalis muscle. As there is a deformation in the shape of the adjacent ear canal due to the expansion and contraction of the temporalis muscle, this change can be measured optically and noninvasively using the ear sensor, to detect mouth breathing. Additionally, when the subject nods, the ear sensor to which the weight is attached shakes. As there is a change in the ear sensor measured values in accordance with this shaking, it can detect the nods of the subject.

Figure 3 shows the measurement principles of the ear sensor. The ear sensor has a miniature optical sensor QRE1113 (Fairchild Semiconductor International Inc., California, USA) and weight SWAROVSKI #5810 (D. Swarovski KG, Wattens, Austria) attached. The weight is a crystal glass sphere with a diameter of 10 mm and a weight of 1.5 g. The weight is connected to the main body of the ear sensor through an acrylic cylinder with a diameter of 1 mm and a length of 10 mm. Built in to the optical sensor are one infrared LED (940 nm) and one phototransistor. The infrared light is irradiated onto the epidermis of the ear canal using the LED, and as the phototransistor receives the reflected light, it measures the movement of the ear canal and shaking of the ear sensor. In concrete terms, when you breathe through the mouth, the movement of opening and closing of the mouth is achieved through the expansion and contraction of the temporalis muscle. As there is a deformation in the shape of the adjacent ear canal with the expansion and contraction of the temporalis muscle, this change can be measured optically and noninvasively using the ear sensor, to detect mouth breathing. Furthermore, by the subject moving their head or the center of gravity of their upper body, the ear sensor with the weight attached shakes. As the ear sensor measurement value changes in accordance with this shaking, it is able to detect movement in the head and body of the subject. That is to say, it is possible to detect MN with one sensor. The weight of this ear sensor is 3.3 g.

The analog signal measured by the ear sensor is converted to a digital signal using the AD converter shown in Figure 2, with a sampling frequency of 2.5 Hz and resolution of 12 bits. The converted signal is both sent to the judging device and stored in memory. The judging device judges that “there was an MN” if the variation in the measurement value from the ear sensor is above a certain *threshold*, and then the actuator is actuated. This *threshold* can be finely tuned using the threshold adjust knob. The algorithm used by the judgment is discussed in Section 2.2.

The timing teaching LED in Figure 2 teaches the timing of mouth breathing and nods to the subject, in the evaluation tests described in Chapter 3, and consists of one blue LED.

In this study, the four elements of AD converter, memory, timing teaching LED, and judging device are realized within one microcomputer, the mbed LPC1768 (Switch Science Inc., Tokyo, Japan), and in-house software (C language). Although this is not shown in Figure 2, a tablet terminal Surface Pro 3 (Microsoft Corp., Washington, USA) was connected via a USB cable to mbed LPC1768, and communication was carried out based on the RS-232C standard; the contents of memory were simultaneously saved on the tablet terminal. The communications software used was CoolTermWin Ver. 1.4.7 (Freeware).

For the threshold adjust knob, the potentiometer RV16YN15SB103 (Tokyo Cosmos Electric Co., Ltd., Kanagawa, Japan) was used.

The neck belt used Free band CP-01 (Kuraray Fastening Co., Ltd., Osaka, Japan). The actuator attached to the neck belt used a linear drive actuator LD 14-002 (Nidec Copal Co., Ltd., Tokyo, Japan). The size of the actuator was 11.2 × 14.0 × 2.6 mm, and its mass was 1.6 g. Additionally, the vibration stroke of the actuator was ±1 mm. The power voltage of the actuator was DC5V. The mass of the neck belt was 15.7 g. We used TB6612FNG (Toshiba Corp., Tokyo, Japan) as the driver IC for the actuator. The driver IC is connected to the tip of Cable 2.

Earable ZEN can be started or stopped using the start/stop button. For the start/stop button, the tactile switch B3F-1050 (OMRON Corp., Kyoto, Japan) was used.

### 2.2. Algorithm

We shall now explain the algorithm for the judging device. This algorithm obtains the variation Δ*v*_*i*_ within 0.4 seconds from the measurement value obtained from the ear sensor, and where this variation Δ*v*_*i*_ exceeds the *threshold* set in advance for each subject, it judges that “an MN occurred” and activates the actuator attached to the neck sensor, applying a slight vibration of 150 Hz for 2 seconds to the left side of the subject's neck. The judging device judges the variation Δ*v*_*i*_ of the measured value during the 2.0 seconds from starting the actuator operation to be 0. This is because it is envisaged that when the actuator is activated and a vibration is applied to the left side of the subject's neck, the subject will be surprised by the vibration and, as a result, breathe through their mouth or move their face or body.

**Figure 4:**
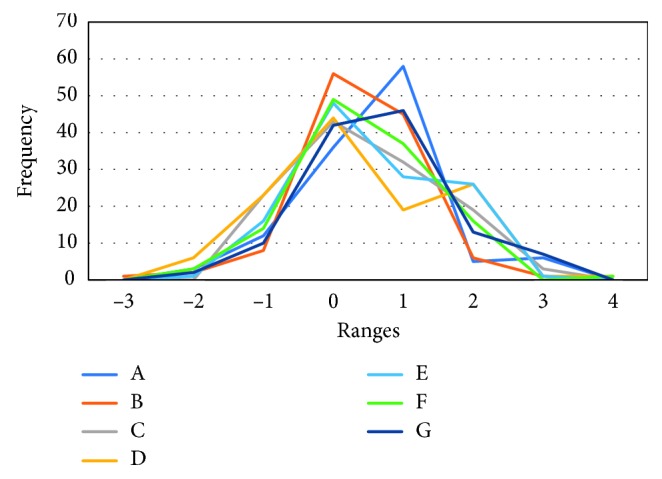
This is the frequency polygon for each subject used in the *threshold* calculation. To organize the class values for each subject when creating the frequency polygon, normalization was performed on the data of each subject used in the *threshold* calculation, according to Equation (5). Additionally, as the number of data items was 120, the number of classes was set to 8 based on Equation (6). The classes were set to −3, −2, −1, 0, 1, 2, 3, 4. For subjects other than subject B, all of the 120 data items for each subject were contained within the class in the diagram. As subject B had one data item within the −4 class, the figure is drawn based on 119 data items.

The *threshold* is defined as in Equation (1). When obtaining this *threshold*, following the experiment content of Section 3.2, the state without MN is maintained in the subject, a measurement occurs every 0.4 seconds in the ear sensor during this time, and this value is converted to the digital signal. The measurement time is set to 48.4 seconds, and a total of 121 converted values *v*_*i*_(*i*=1,2,3,…121) are recorded. The difference (*v*_2_ − *v*_1_, *v*_3_ − *v*_2_, *v*_4_ − *v*_3_,…*v*_121_ − *v*_120_) in the AD converted values *v*_*i*_ are obtained every 0.4 seconds, and these are set to *x*_*i*_ (*i* = 1, 2,3,…, 120). The number of *x*_*i*_ data items *n* is 120. The average value of *x*_*i*_ is set to $\bar{x}$. *Threshold* is the definition presumed when *x*_*i*_ is a normal distribution. The distribution of *x*_*i*_ measured values is shown in Section 4.1 (Figure 4). *F*_t_ is the coefficient for adjusting the *threshold*, and this can be set within the range of 0.5 to 2.0 using the threshold adjust knob in Figure 2. The *threshold* is defined as in Equation (1), using the estimated values for the population mean and population variance for the 99% confidence interval obtained in Equations (2) and (3), based on 120 items of data *x*_*i*_ when the earable ZEN is actually used. Thus, even if the user is not doing MN, in rare cases, this may be above the *threshold* value. Therefore, when the earable ZEN is actually operated, it is necessary to finely tune *F*_t_ using the threshold adjust knob so that variation Δ*v*_*i*_ does not exceed the *threshold* when MN is not taking place and exceeds the *threshold* when MN is taking place:

(1) $$\begin{array}{c} threshold=F_{\text{t}}\left(\mu_{\max}+4\sigma_{\max}\right). \end{array}$$

Here, with *σ*_max_ as the maximum value of the population variance estimated value within the 99% confidence interval, this is obtained from Equation (2). Furthermore, with *μ*_max_ as the maximum value of the population mean estimated value within the confidence interval, this is obtained from Equation (3):

(2) σmax=s^χ0.9752n−1  ,

where

(3) s^=∑i=1nxi−x¯2  ,xi=vi−vi+1,  μmax=x¯+2.  58σmaxn.

Variation Δ*v*_*i*_ is expressed as the absolute value of the difference between the AD converted value of value *v*_*i*_, currently measured in the ear sensor, and AD converted value of value *v*_*i*+1_, measured 0.4 seconds earlier in the ear sensor:

(4) $$\begin{array}{c} \Delta v_{i}=\left|x_{i}\right|. \end{array}$$

## 3. Evaluation Experiments

### 3.1. Subjects

The subjects of the evaluation experiments in Section 3.2 and Section 3.3 were seven healthy subjects (males and females between 22 and 62 years old, mean age 40.6 years old). These were referred to as A, B, C, D, E, F, and G, respectively. The subjects attached the ear sensors to their ears, without any concept of big or small. They did not recognize any symptoms of ear pain or fatigue in themselves, and those currently receiving medical treatment were excluded. Additionally, in the same way, the neck belts were worn without any concept of big or small. They did not recognize any symptoms of neck pain or fatigue in themselves, and those currently receiving medical treatment were excluded. Any subjects who felt discomfort due to wearing the ear sensor and neck belt were excluded from the group of subjects.

This study received the approval of the “Shinshu University Ethics Committee for Studies Aimed at Humans,” and the consent to participate in the study was received from the subjects after a full explanation was provided. In all of the experiments, the ear sensor and neck belt, for hygienic reasons, were cleaned with a clean brush before and after use and disinfected with disinfecting ethanol.

### 3.2. Measurement of Data for Creating the Threshold

The subject was asked to perform zazen while wearing the earable ZEN (ear sensor on the left ear of the subject and neck belt attached to the neck). During zazen, the subject was asked to perform abdominal respiration, breathing slowly in through the nose and breathing out from the nose. They were also asked to keep their eyes half open, staring 1 m ahead. Additionally, they were asked to sit in a posture where they placed their right foot on their left thigh and their left foot on their right thigh, relaxed their shoulders, and stretched their back. Those subjects who were unable to place their feet on their thighs were asked to perform the experiment sitting on a chair so that their body would not be in pain. The time spent performing zazen was set to one minute, and the 48.4 seconds of data measured from 5 seconds to 53.4 seconds (121 units) was recorded to the memory. The subjects were asked to perform zazen while taking adequate care not to breathe through their mouths (only breathe through their noses) and not to move their face or bodies. The measurement during this experiment was started by pressing the start/stop button, and it ended automatically after 48.4 seconds had passed. It was also possible to end the measurement by pressing the start/stop button.

### 3.3. Earable ZEN Evaluation Experiments

In this evaluation experiment, we evaluated whether it was possible for earable ZEN to correctly detect MN in the subjects. Specifically, the subjects were asked to perform zazen twice while wearing the earable ZEN for approximately 3 minutes (187.2 seconds, sampling frequency 0.4 sec, number of samples: 468). During the first zazen, they were asked to breathe deeply through the mouth 10 times in accordance with the lighting of the timing teaching LED. During the second zazen, they were asked to nod 10 times in accordance with the lighting of the timing teaching LED.

In this experiment, the *threshold* for each subject obtained from the experiment in Section 3.2 was set in the memory of the experiment device in advance. In this experiment, the coefficient for adjusting the *threshold*, *F*_t_, was set to 1.0. Every time a measurement occurred with the ear sensor (every 0.4 seconds), when it was judged by the judging device that there was MN, based on the algorithm in Section 2.2, the actuator attached to the neck belt was activated. A slight vibration of 150 Hz was then applied to the left side of the neck of the subject for a period of 2 seconds. The timing teaching LED flashing state, ear sensor measurement values, and judgment results were recorded to the tablet terminal connected to the microcomputer device in Figure 1.

The measurement in this experiment was started by pressing the start/stop button and stopped automatically after 187.2 seconds. It was also possible to stop the measurement by pressing the start/stop button during measurement.

## 4. Results

### 4.1. Data Measurement Results for the Purpose of Creating the Threshold

The *threshold* for each subject was calculated based on the measurement results obtained through the experiment in Section 3.2 according to the method in Section 2.2. The calculated results are shown in Table 1. The variation when mouth breathing and variation in nods in Table 1 are explained in Section 4.2. Figure 4 shows the frequency polygon for the data of each subject used in the *threshold* calculation in Table 1. To organize the class values for each subject created in Figure 4, normalization was performed on the data *x*_*i*_ for each subject used in the *threshold* calculation based on Equation (5). By performing normalization, the mean value is set to 0, and the variance is set to 1. Additionally, with the number of classes *k*, based on Equation (6) (Sturges' formula), as the number of *x*_*i*_ data items *n* is 120, this is set to 8, and the classes are set to −3, −2, −1, 0, 1, 2, 3, and 4.

As shown in Table 1, the mean value of the *threshold* for the seven subjects was 2.96 × 10^–3^ (1.82 to 4.32 × 10^–3^). Compared to the mean value, subjects A, E, and G had larger values, and subjects C and F had small values. The peaks for each test subject in Figure 4 were in class 1 for subjects A and G, while the other subjects were in class 0. Additionally, for all subjects other than subject B, the 120 data items for each subject used in calculating the *threshold* were all within the class of −3 to 4. Subject B had one data item in the −4 class. When the *threshold* was recalculated with the data item in −4 for subject B excluded as an outlier, this was 2.49 × 10^−3^. When included, this was 2.68 × 10^−3^, as shown in Table 1:

(5) $$\begin{array}{c} z_{i}=\frac{x_{i}-\bar{x}}{\sigma }, \end{array}$$

where

(6) $$\begin{array}{c} \sigma =\sqrt{\frac{\hat{s}}{n}},\\ k\approx \;1+\log_{2}\;\;\left(n\right). \end{array}$$

### 4.2. Earable ZEN Evaluation Experiment Results

**Figure 5:**
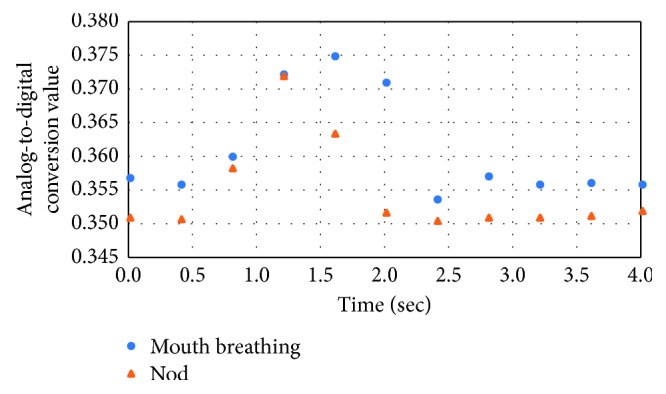
Measurement results (AD converted values) when subject A performs mouth breathing and nods. This is an inverted U waveform showing the signal increasing and decreasing for both mouth breathing and nods. This same trend in the waveform was seen for all subjects other than subject A.

Tables 1 and 2 and Figure 5 show the earable ZEN evaluation experiment results obtained using the method in Section 3.3.

The variation for mouth breathing and the variation for nods for each subject are shown in Table 1. The variation for mouth breathing for each subject was obtained by the subject performing mouth breathing 10 times and seeking the Δ*v*_*i*_ maximum value each time, with the mean of the maximum value over 10 times being taken. The variation when nodding was obtained in the same way.

In Figure 5, the measurement values (AD converted values) when subject A performs mouth breathing or nods is plotted by time on the horizontal axis. There is an inverted U waveform showing the signal increasing and decreasing for both mouth breathing and nods. The tendency in this waveform can be seen in the same way for subjects B to G. Additionally, when the mouth breathing value in Table 1 is large, the variation in the amplitude of the measurement waveform in Figure 5 is also large. The tendency seen in mouth breathing is also the same for nods.

The *accuracy* of Table 2 is obtained using Equation (7). The *accuracy* becoming closer to 1 indicates the greater extent to which the earable ZEN is detecting MN correctly:

(7) $$\begin{array}{c} accuracy=\frac{\text{TP }+\text{ TN}}{\text{TP }+\text{ FP }+\text{ FN }+\text{ TN}}, \end{array}$$

where TP (true positive) indicates the number of times the judging device judges MN when the subject performs MN (number at the *threshold* or above) and FP (false positive) indicates the number of times the judging device judges MN despite the fact that the user has not performed MN. TN (true negative) is the number of times that the judging device did not judge MN when the subject did not perform MN (times below *threshold*) and FN (false negative) is the number of times the judging device did not judge MN despite the fact that the subject performed MN. The *precision* and *recall* in Table 2 can also be obtained from the following equations:

(8) $$\begin{array}{c} precision=\frac{\text{TP}}{\text{TP }+\text{ FP }}, \end{array}$$

(9) $$\begin{array}{c} recall=\frac{\text{TP}}{\text{TP }+\text{ FN }}, \end{array}$$

where *precision* represents the ratio of the number of times the judging device correctly judges a movement as MN to the number of times that the judging device judges a movement as MN. On the contrary, *recall* represents the ratio of the number of times that the judging device correctly judges a movement as MN to the number of times the subject actually performs MN.

As shown in Table 2, the mean value for *accuracy* in the seven subjects calculated from the evaluation experiment results was 0.999 for mouth breathing and 1.000 for nods. In the same way, the mean value for *precision* was 0.987 for mouth breathing and 1.000 for nods, with the mean value for *recall* being 0.971 for mouth breathing and 1.000 for nods. For subjects A, D, E, and G (4 out of the 7 subjects), *accuracy*, *precision*, and *recall* were all 1.000.

In all of the experiments, none of the subjects felt that the ear sensor or neck belt were an obstacle to zazen. Additionally, none of the subjects felt that the shaking of the actuator was unpleasant. Furthermore, none of the subjects felt fatigue as a result of the experiment.

## 5. Discussion

The *recall* for mouth breathing for subjects C and F in Table 2 was 0.90. For the other subjects, this was 1.00. The fact that recall was 0.90 expressed the fact that 10% of the case in which the subject actually performed mouth breathing was not judged by the judging device to be mouth breathing. The differences between the subjects C and F, and the other subjects, were that the mouth breathing and *threshold* value ratios in Table 1 were small. Subject C was 1.6 (= mouth breathing value ÷ *threshold* value), and subject F was 1.4. The other subjects were from 2.5 to 6.6. Additionally, subjects C and F had a smaller *threshold* than other subjects. That is to say, for subjects C and F, the movement of the ear canal can be said to be comparatively smaller than the other subjects. The nod values of subjects C and F were virtually the same as that of other subjects. The nod values are determined by the extent to which the weight shakes at the time of the nod. The shaking of the weights of subjects C and F were not seen to have the same difference with other subjects as was seen in the values for mouth breathing. Even when the ratio of the values to *threshold* were low, as seen in mouth breathing for subjects C and F, *recall* with the current method had an *accuracy* of 0.90, and this was considered to be a high score. However, for the practical use of earable ZEN, the aim is for *recall* to be 1.00, and the goal will be to improve the ear sensor and algorithm in the future.

The *precision* of subject B when mouth breathing was 0.91 in Table 2. This was because, in spite of the case that subject B was neither mouth breathing nor nodding, the judging device mistakenly judged that “mouth breathing occurred” once during 408 measurements. Here, 408 times refers to the number of measurements when subject B was neither mouth breathing nor nodding. For these times, the *recall* of subject B in Table 2 is 1.00; and thus, of the total number of 468 measurements taken during one experiment, we can see that MN occurred 10 times, and when subtracting those 10 times from the 468 times, with the measurement value Δ*v*_*i*_ for the 5 times just after the judgment of MN as 0, MN is not judged during 0 (this has no effect on *precision*). Therefore, if we further subtract the 50 measurement times for which the 10 MN times occurred, this becomes 408 times. In other words, the *precision* of subject B when mouth breathing of 0.91 means that when subject B was neither mouth breathing nor nodding, values above the *threshold* were measured with a probability of 0.25% (1 time ÷ 408 times). The actual measurement value Δ*v*_*i*_ at this time was 2.69 × 10^−3^, exceeding the *threshold* 2.68 × 10^−3^ by 0.01 × 10^−3^. This can be resolved by finely tuning the *threshold* using the threshold adjust knob. Additionally, in the case of subject B only, one of the 120 data items used in the *threshold* calculation did not fall within the class of −3 to 4. This was considered to be an outlier, and when the *threshold* was calculated without using outliers, this became 2.49 × 10^−3^ and, using this *threshold*, *precision* became 1.00.

As shown in Figure 4, normal distribution was performed on the data groups of each subject used to obtain the *threshold*. In Section 2.2, it is presumed that the *threshold* is a normal distribution, and this can be said to be correct.

As shown in Figure 5, for both mouth breathing and nodding, the signals increase and decrease causing an inverse U-shaped waveform, and the two waveforms are similar in shape. This expresses the fact that with the current measurement method, there is no distinction between mouth breathing and nods. The results of Figure 5 also suggest that the ear sensor could be used to measure mouth breathing if the weight was removed.

As a next step, we would like to conduct a more accurate evaluation experiment using a spirometer or a nasal respiration-measuring device.

As, in this way, consistency was seen in the measurement results of the small number of 7 test subjects with different genders and ages, sufficient investigation may be possible even with this number.

This study focused only on the use of zazen. Zazen involves sitting cross-legged and not moving your body while abdominal breathing, slowly and deeply, through your nose. That is to say, in zazen, you adopt a special posture that you would not use in everyday life. The earable ZEN developed in this study checks that the signals measured in the ear sensor are contained below the *threshold* value for the subject in question while they are adopting the special zazen posture and that there are no disturbances in their breathing or posture. Previously, we have seen studies [25–31] that measure the chewing amount and tongue movement using the ear sensor, but these were studies that estimated “what the subject has done” based on changes in the shape of the ear canal. However, the focus of the approach in this study is different to that of previous studies, in which it estimates whether “the subject is not doing anything (is sitting still)”.

We focus on zazen as a method of relieving daily mental fatigue. We have researched and developed this earable ZEN as a wearable system that supports individuals so they can perform zazen casually and simply at home, without going to a temple and without a zazen monitor being present. With earable ZEN, it is necessary to attach an earphone-type sensor to your ear when performing zazen and wind a belt around your neck. This may give some people an odd sensation. However, we considered that when introducing zazen into everyday life, it was important to propose a way of being able to flexibly change clothes and equipment during zazen, while diligently following the essentials of zazen, that is, “prepare your body (posture), your breathing, and your mind (spirit).” The earable ZEN can be considered to be one such proposal. Moreover, in the last 40 years or so, enjoying music through your earphones during everyday life has become socially acceptable, and the earable ZEN is an extension of the same. Attaching a speaker to the earable ZEN ear sensor also makes it possible to listen to music while performing zazen. Additionally, attaching ear sensors with speakers to both ears should make it possible to output antiphase noise in relation to the surrounding noise from the speakers, so that surrounding noise cannot be heard. In the future, we need to look one by one at the aspects that need to be considered when actually operating earable ZEN, such as culture, customs, and manners. Moreover, we will analyze the meditative states using an EEG and the measurement results obtained from earable ZEN to clarify the relationship between the two as well as use those results to improve earable ZEN. Furthermore, we will investigate adding an EEG sensor to the earable ZEN to measure meditative states.

We aim to improve earable ZEN by resolving the above tasks, and with experiments under variety of conditions considered for zazen, increase the practicability of earable ZEN.

## 6. Conclusions

We developed the earable ZEN as the prototype for a wearable system that would allow people to perform zazen casually and simply at home, without visiting the temple, and without a monitor being present. During zazen, it is essential to prepare your breathing and posture. During zazen, you sit still, without moving your head or body, while breathing slowly and deeply through your nose. The earable ZEN has functions to monitor whether the person performing zazen (user) accidentally breathes through their mouth or yawns (disturbances in breathing) or drops off to sleep or moves their head (disturbances in posture) and communicates this to the user. In particular, earable Zen detected incorrect breathing, “performing deep mouth breaths (not breathing through the nose)” and incorrect posture “nodding (dozing)” when conducting zazen. This prototype does not measure meditative states.

In this paper, we show the mechanism of the earable ZEN and the results of the evaluation experiments. The earable ZEN is comprised of earphone-type ear sensor to measure disturbances in breathing or posture, a microcomputer to judge breathing or posture disturbances from these measured values, and the neck belt-type actuator to communicate the results to the user using vibrations. In the evaluation experiments, seven healthy subjects, A to G (males and females from 21 years old to 62 years old, average age 40.6 years old), wearing the earable ZEN, performed zazen twice for approximately three minutes (187.2 seconds). The subjects were asked to breathe deeply 10 times during the first zazen, in accordance with the timing teaching LED. This was used to simulate disturbances in breathing as “deep mouth breathing,” with mouth breathing and yawning considered as accidental breathing disturbances. During the second zazen, the subjects were asked to nod 10 times. This was used to simulate disturbances in posture caused by nodding off and shaking of the head, with “nodding” considered as a posture disturbance. The results of the evaluation experiments were that the mean value for *accuracy* in the seven subjects calculated from the evaluation experiment results was 99.9% for mouth breathing and 100% for nods. In the same way, the mean value for *precision* was 98.7% for mouth breathing and 100% for nods, and the mean value for *recall* was 97.1% for mouth breathing and 100% for nods. In this experiment, no subjects felt that the ear sensor or neck belt were an obstacle to zazen. Additionally, none of the subjects felt that the vibration from the actuator was unpleasant. Furthermore, none of the subjects felt fatigue during the experiment.

As next steps, by performing a comparative analysis of meditative states analyzed using an EEG and the results of measurement obtained from the earable ZEN, we wish to clarify the relationship between the two while improving the earable ZEN based on those results.

While looking one by one at the aspects that need to be considered when actually operating earable ZEN, such as culture, customs, and manners, we aim to improve earable ZEN with experiments and a variety of conditions considered for zazen and thus increase the practicability of earable ZEN.

## Figures and Tables

**Table 1:** The *threshold*, variation when mouth breathing, and variation when nodding for each subject. The *threshold* for each subject was calculated using the measurement values acquired from the experiment in Section 3.2 with the method in Section 2.2. The variation for mouth breathing for each subject was obtained by the subject performing mouth breathing 10 times, seeking the Δ*v*_*i*_ maximum value each time, with the mean of the maximum value over 10 times being taken. The variation when nodding was obtained in the same way.

Subject	A	B	C	D	E	F	G	Average
*Threshold*	3.85 × 10^−3^	2.68 × 10^−3^	1.96 × 10^−3^	2.43 × 10^−3^	4.32 × 10^−3^	1.82 × 10^−3^	3.67 × 10^−3^	2.96 × 10^−3^
Mouth breathing	18.00 × 10^−3^	14.43 × 10^−3^	5.16 × 10^−3^	15.12 × 10^−3^	10.57 × 10^−3^	3.90 × 10^−3^	20.90 × 10^−3^	12.58 × 10^−3^
Nod	11.97 × 10^−3^	17.61 × 10^−3^	11.84 × 10^−3^	12.75 × 10^−3^	10.57 × 10^−3^	9.26 × 10^−3^	12.14 × 10^−3^	12.30 × 10^−3^

**Table 2:** Evaluation experiment results from the earable ZEN: the seven healthy subjects A to G wearing the earable ZEN (males and females aged 21 to 62 years old, mean age 40.6 years old) performed zazen twice for approximately 3 minutes (187.2 seconds). During the first zazen, the subjects breathed 10 times deeply through the mouth, in accordance with the timing teaching LED. This simulated disturbances in breathing. During the second zazen, nods were performed 10 times, in accordance with the timing teaching LED. This simulated disturbances in posture. From the measurement results from the earable ZEN judging device during the experiment (values obtained in the experiment using the method in Section 3.3), *accuracy*, *precision*, and *recall* were obtained using Equations (7)–(9) in Section 4.2.

Subject	Item	*Accuracy*	*Precision*	*Recall*
A	Mouth breathing	1.00	1.00	1.00
	Nod	1.00	1.00	1.00

B	Mouth breathing	1.00	0.91	1.00
	Nod	1.00	1.00	1.00

C	Mouth breathing	1.00	1.00	0.90
	Nod	1.00	1.00	1.00

D	Mouth breathing	1.00	1.00	1.00
	Nod	1.00	1.00	1.00

E	Mouth breathing	1.00	1.00	1.00
	Nod	1.00	1.00	1.00

F	Mouth breathing	1.00	1.00	0.90
	Nod	1.00	1.00	1.00

G	Mouth breathing	1.00	1.00	1.00
	Nod	1.00	1.00	1.00

Average	Mouth breathing	0.999	0.987	0.971
	Nod	1.000	1.000	1.000

## Data Availability

The measurement data used to support the findings of this study are restricted by the “Shinshu University Ethics Committee for Studies Aimed at Humans” in order to protect subject privacy. Data are available from authors for researchers who meet the criteria for access to confidential data.

## References

[1] Marchand W. R. (2012). Mindfulness-based stress reduction, mindfulness-based cognitive therapy, and zen meditation for depression, anxiety, pain, and psychological distress. *Journal of Psychiatric Practice*.

[2] Chiesa A., Serretti A. (2009). A systematic review of neurobiological and clinical features of mindfulness meditations. *Psychological Medicine*.

[3] Ospina M. B., Bond K., Karkhaneh M. (2008). Clinical trials of meditation practices in health care: characteristics and quality. *Journal of Alternative and Complementary Medicine*.

[4] Suzuki D. T. (1934). *An Introduction to Zen Buddhism*.

[5] Suzuki S. (1970). *Zen Mind, Beginner’s Mind*.

[6] Nitobe I. (2002). *Bushido: The Soul of Japan*.

[7] Buksbazen J. D. (2002). *Zen Meditation in Plain English*.

[8] Hoshiyama M., Hoshiyama A. Heart rate variability associated with different modes of lower abdominal muscle tension during Zen meditation.

[9] Hoshiyama M., Hoshiyama A. Heart rate variability associated with different modes of respiration during Zen meditation.

[10] Hoshiyama M., Hoshiyama A. Repeatability value in heart rate associated with experienced Zen meditation.

[11] Hoshiyama M., Hoshiyama A. Heart rate variability associated with experienced Zen meditation.

[12] Miguel A., González G., Juan J., Castro R., Fernández-Chimeno M. A novel index based on fractional calculus to assess the dynamics of heart rate variability: changes due to Chi or Yoga meditations.

[13] Cysarz D., Büssing A. (2005). Cardiorespiratory synchronization during Zen meditation. *European Journal of Applied Physiology*.

[14] Baglio S., Bucolo M., Fortuna F., Frasca M., La Rosa M., Shannahoff-Khalsa D. S. MEG signals spatial power distribution and gamma band activity in yoga breathing exercises.

[15] Fortuna L., Bucolo M., Frasca M. Independent component analysis of magnetoencephalography data.

[16] Bucolo M., Grazia F. D., Sapuppo F., Shannahoff-Khalsa D. Identification of MEG-related brain dynamics induced by a yogic breathing technique.

[17] Kasamatsu A., Okuma T., Takenaka S., Koga E., Ikeda K., Sugiyama H. (1957). The EEG of zen and yoga practitioners. *Electroencephalography and Clinical Neurophysiology Supplement*.

[18] Hirai T., Izawa S., Koga E. (1959). EEG and zen buddhism. *Electroencephalography and Clinical Neurophysiology Supplement*.

[19] Hirai T. (1960). Electroencephalographic study on the Zen meditation (Zazen)-EEG changes during the concentrated relaxation. *Folia Psychiatrica et Neurologica Japonica*.

[20] Kasamatsu A., Hirai T. (1966). An electroencephalographic study on the Zen meditation (Zazen). *Psychiatry and Clinical Neurosciences*.

[21] Kasamatsu K., Hirai T., Tart A. C. (1969). An electroencephalographicstudyonthezen (Zazen). *Altered states of consciousness*.

[22] Murata T., Koshino Y., Omori M. (1994). Quantitative EEG study on zen meditation (zazen). *Psychiatry and Clinical Neurosciences*.

[23] Faber P. L., Lehmann D., Gianotti L. R. R. (2014). Zazen meditation and no-task resting EEG compared with LORETA intracortical source localization. *Cognitive Processing*.

[24] Phongsuphap S., Pongsupap Y., Chandanamattha P., Lursinsap C. (2008). Changes in heart rate variability during concentration meditation. *International Journal of Cardiology*.

[25] Taniguchi K., Kondo H., Kurosawa M., Nishikawa A. (2018). Earable TEMPO: a novel, hands-free input device that uses the movement of the tongue measured with a wearable ear sensor. *Sensors*.

[26] Taniguchi K., Kondo H., Tanaka T., Nishikawa A. (2018). Earable RCC: development of an earphone-type reliable chewing-count measurement device. *Journal of Healthcare Engineering*.

[27] Taniguchi K., Chiaki H., Kurosawa M., Nishikawa A. (2017). A novel earphone type sensor for measuring mealtime: consideration of the method to distinguish between running and meals. *Sensors*.

[28] Taniguchi K., Kurosawa M., Nishikawa A. Earable: Wearable ear computer.

[29] Kurosawa M., Taniguchi K., Nishikawa A. A basic study of occlusal force measured using a wearable ear sensor.

[30] Taniguchi K., Horise Y., Nishikawa A., Iwaki S. A novel wearable input device using movement of ear-canals. TBIS2012.

[31] US Patent No. US8994647 (2015). *Input Device, Wearable Computer, and Input Method*.

